# Goat hair as a bioindicator of environmental contaminants and adrenal activation during vertical transhumance

**DOI:** 10.3389/fvets.2023.1274081

**Published:** 2023-11-09

**Authors:** Stella Agradi, Albana Munga, Olimpia Barbato, Rupert Palme, Duygu Tarhan, Bengü Bilgiç, Banu Dokuzeylül, Alev Meltem Ercan, Mehmet Erman Or, Gabriele Brecchia, Giulio Curone, Susanna Draghi, Daniele Vigo, Maria Laura Marongiu, Marta González-Cabrera, Laura Menchetti

**Affiliations:** ^1^Department of Veterinary Medicine and Animal Sciences, University of Milan, Lodi, Italy; ^2^Faculty of Veterinary Medicine, Agricultural University of Tirana, Tirana, Albania; ^3^Department of Veterinary Medicine, University of Perugia, Perugia, Italy; ^4^Unit of Physiology, Pathophysiology, and Experimental Endocrinology, Department of Biomedical Sciences, University of Veterinary Medicine of Vienna, Vienna, Austria; ^5^Department of Biophysics, Cerrahpasa Faculty of Medicine, Istanbul University-Cerrahpasa, Istanbul, Türkiye; ^6^Department of Biophysics, School of Medicine, Bahcesehir University, Istanbul, Türkiye; ^7^Department of Internal Medicine, Faculty of Veterinary Medicine, Istanbul University-Cerrahpasa, Istanbul, Türkiye; ^8^Department of Veterinary Medicine, University of Sassari, Sassari, Italy; ^9^Institute of Animal Health and Food Safety, Universidad de Las Palmas de Gran Canaria, Arucas, Spain; ^10^School of Biosciences and Veterinary Medicine, University of Camerino, Matelica, Italy

**Keywords:** autochthonous breed, small ruminant, alpine pasture, bioindicator, heavy metals, trace elements, animal welfare, hair cortisol

## Abstract

Autochthonous breeds of livestock are considered a pivotal genetic resource for agriculture, rural development, and food and nutrition security. In the Italian Alps, local livestock breeds are maintained using the traditional alpine farming system based on vertical transhumance, with the use of alpine pastures from late spring to autumn and indoor housing with a hay-based diet for the remaining part of the year. Because of their tight link with the territory of origin, local breeds could be used to biomonitor environmental contaminations. Moreover, animal welfare should also be monitored during transhumance in animals, which are exposed to a sudden farming system change and different types of stressors. For these reasons, this investigation hypothesized that the content of trace elements, heavy metals, and cortisol in the hair of goats changes during vertical transhumance, possibly reflecting different dietary contents and activity of the hypothalamic–pituitary–adrenal (HPA) axis. This study aimed to assess the response of an Italian local goat breed to the change from indoor housing to alpine pasture in summer in terms of hair concentrations of (i) trace elements and heavy metals and (ii) cortisol. The regrown hair of Frisa goats was monthly collected for 2 consecutive years (*n* = 10 for heavy metals and trace elements and *n* = 6 for cortisol in 2021, *n* = 17 for both analyses in 2022), once before vertical transhumance and twice after that event. Hair was then analyzed for trace elements, heavy metals, and cortisol by inductively coupled plasma-optical emission spectrophotometer (ICP-OES) and enzyme immunoassay (EIA), respectively. Data were analyzed by multilevel models. The results showed an increase in As content during alpine pasture (*p* < 0.01), probably reflecting the soil and water As contents of the grazing area, while Mg, Zn, and Al (*p* < 0.01) followed the opposite trend, decreasing in the second month after vertical transhumance. Hair cortisol concentrations increased during 2 months of alpine pasture (*p* < 0.001), indicating an increase in the activation of the HPA axis, in agreement with previous studies. Future investigations can consider a longer study period and the development of *ad hoc* animal welfare indicators.

## Introduction

Since the first livestock animal domestication events ~12, 000 years ago, pastoralism has spread across the world following human migrations (1). Even in the most complex sedentary farming societies, the need for a continuous source of feed during the year has led to the practice of transhumance. Specifically, for those human populations living near mountainous regions, vertical transhumance still represents a fundamental strategy to guarantee quality grazing through the different seasons due to the handling of the flocks and herds along an altitudinal gradient (2), particularly true when considering the Alpine region of Italy. However, despite the socioecological importance of vertical transhumance and its related supply of ecosystem services, the practice is gradually disappearing (3).

The maintenance of the traditional semi-extensive farming system of autochthonous goats, sheep, and cattle breeds in Northern Italy, which provides a hay-based diet with indoor housing in winter and fresh forages from late spring to autumn through alpine pastures (4, 5), also plays a useful role in a One-Welfare perspective. Indeed, this system generates a close bond of interdependence between the environment in which these livestock animals are traditionally bred for centuries, if not millennia, and the local breed itself. This leads to the opportunity to use these animals as bioindicators, indicating harmful changes caused by pollution in the surrounding ecosystem (6), also due to the ease with which observations or biological samples can be obtained. Thus, recently, the use of autochthonous livestock breeds to monitor the levels of environmental trace elements and heavy metals is growing. These types of molecules could be toxic at specific concentrations and represent a risk to human, animal, and environmental health (7, 8). Herbivores are constantly exposed to their uptake *via* ingestion of small amounts of soil, polluted vegetation, and water. It must, however, be taken into consideration that species-specific physiological characteristics (9), as well as seasonal, ecological, and ethological variations, may affect exposition and absorption (10, 11). Regarding the human part, the food chain pathway is considered one of the major pathways of exposition (12), and, also for this reason, controlling the possible contamination of products of animal origin, such as cheese or meat, is necessary.

Another aspect that is positively linked to the alpine pasture is animal welfare. Studies have indicated that both consumers and farmers consider livestock access to pasture important because of the shared idea that allowing animals to express their natural behavior is pivotal (13). The European Union encourages these practices through the provision of specific CAP (Common Agricultural Policy) funds. The general aim is to counteract the abandonment of mountain farming areas which, if appropriately used and managed, can contribute to ensure real protection of the territory, biodiversity, prevention of hydrogeological risk, and soil erosion, and, consequently, adaptation to climate change (4, 14). However, the action of grazing also involves other aspects that affect animals and need to be considered, such as feed quality and availability, predation, parasitosis, environmental conditions, and biosecurity for transmissible diseases between flocks/herds and wild animals (15). Therefore, animal welfare must be monitored also in grazing animals. A classic welfare indicator is cortisol (16), the end-product of the activation of the hypothalamic–pituitary–adrenal (HPA) axis, which is released in response to different types of stimuli (i.e., stressors). The actions of this hormone system are tightly regulated to ensure that the body can respond quickly to the stressors, adjust to these challenges, and recover to the original homeostasis (16, 17).

A useful tool to evaluate trace elements, heavy metals, and cortisol in animals is the analysis of hair. Hair is a metabolically inert biological matrix (after it has left the epidermis) and chemically homogeneous (18). It can be easily collected from domestic animals with a non-invasive modality and, due to its growth pattern, allows for biological retrospective imaging of the element being analyzed for a few months from the time of collection. Finally, the sampling could be repeated on the same skin area, allowing a repeated measure. The analysis of hair enables the determination of long-term exposure of both trace elements and heavy metals due to the presence of sulfhydryl group (–SH) of cysteine capable of their chelation (18). On the other hand, hair cortisol concentration is considered a good indicator of the long-term activation of the HPA axis, which is used to evaluate animal stress, welfare, and the ability to cope with environmental challenges (16). The use of the hair for the determination of environmental contaminants and animal stress indicators is in perfect agreement with the One-Welfare principles, which invite a holistic consideration of human health and environmental protection in compliance with animal welfare (19, 20).

We hypothesized that the content of trace elements and heavy metals and cortisol in the hair of goats changes during transhumance, possibly reflecting different dietary contents and activities of the HPA axis, respectively. Thus, this study aimed to assess the response of an Italian local goat breed to the change from indoor housing to alpine pasture in summer in terms of hair concentrations of (i) trace elements and heavy metals and (ii) cortisol. The findings could support the possible use of the hair of local breeds as bioindicators of environmental pollution of trace elements and heavy metals and, at the same time, as a non-invasive tool to evaluate the animal's adaptive response to farming system changes in agreement with the One-Welfare principles.

## Materials and methods

### Animals and hair collection

For this study, 24 female Frisa goats were enrolled. The Frisa or Frontalasca goat is an autochthonous Lombard goat breed native to the Rezzalo Valley (in the Frontale municipality). The Anagraphic Registry was activated in 1997, and at the end of 2016, the registered population was 785 heads in 51 farms (21). It is a double aptitude breed raised with the alpine traditional farming system. The animals were all part of the same flock, which is bred in Val Bregaglia in the alpine area of Lombardy, near the Swiss border, one of the typical and traditional breeding areas. The animals were kept under a traditional semi-extensive farming system, with indoor housing and a hay-based diet at ~650 m.s.l. from November to May (the hay was purchased from Pavia province, South Lombardy) and free grazing on alpine pasture at ~1400–2000 m.s.l. in the remaining part of the year (Supplementary Figures S1, S2). The reproduction occurred by natural mating, and the birth season took place in the last two decades of December. For this reason, the goats enrolled were all at the same lactational and reproductive stages (lactating and not pregnant). The goats were milked twice a day, with a milking machine during the indoor housing and by hand during the alpine pasture period, till the second week of August. The milk was entirely used for cheese production. Before including animals in the study, an anamnestic investigation on the sanitary status of the flock was conducted with the breeder, and a physical examination of all goats was conducted by a team of veterinarians. Only clinically healthy animals were enrolled. The mean body condition score was 2.7/5 at the beginning of the study. Nulliparous and primiparous goats were not enrolled in the study. The mean (and standard deviation) age was 6.2 ± 2.9 years (range: 2.2–12.3 years). The involved animals were sampled for 2 consecutive years (2021 (*n* = 10) and 2022 (*n* = 17) for trace elements and heavy metals and 2021 (*n* = 6) and 2022 (*n* = 17) for cortisol), according to the same experimental plan and the same year. The experimental design was repeated in the same conditions (same farm, same transhumance, and same pastures). The collection of hair samples from live animals was performed with respect to animal welfare according to current legislation. The study was conducted with the approval of the Institutional Animal Care and Use Committee of Università degli Studi di Milano (Permission OPBA_04_2021). Hair samples were collected from the left rump of each animal in a region of ~10 cm^2^ clipped as close to the skin as possible with an electric clipper, according to the “shave–reshave” method, and stored in polyethylene bags at room temperature, protected from sunlight till analysis (22). Hair was shaved the first time before 1 month of vertical transhumance. Then, regrown hair was taken from the same area 1 day before vertical transhumance (before grazing) and 1 month and 2 months after the vertical transhumance. Only regrown hair was analyzed to determine trace elements, heavy metals, and cortisol concentrations because only for this type of sample, the re-growth time was known (30 days). The present protocol is described in detail by Heimbürge et al. (23).

### Hair analysis

Chromium (Cr), copper (Cu), iron (Fe), magnesium (Mg), zinc (Zn), arsenic (As), boron (B), nickel (Ni), lead (Pb), and aluminum (Al) hair concentrations were determined by an inductively coupled plasma-optical emission spectrophotometer (ICP-OES; Thermo iCAP 6000series) at Trace and Toxic Element Analysis Laboratory at Biophysics Department, Faculty of Medicine, Istanbul University-Cerrahpasa (device parameters are presented in Supplementary Table S1, and wavelengths used to determine each element are presented in Supplementary Table S2). In total, 1000 ppm of each element analyzed (Chem-Lab NV, Zedelgem, Belgium) was used to prepare the standard solutions in deionized water. The blank solution was distilled water. These solutions were used to obtain reproducible and linear calibration curves and calculate the correlation coefficient. The analysis was conducted in triplicate. The wet decomposition method for trace element and heavy metal measurements was used to prepare all samples, using 0.04 g per sample. The hair was added to 2 ml 65% HNO_3_ (Merck, Darmstadt, Germany) and 1 ml 60% HClO_4_ (Panreac, Barcellona, Spain) and dissolved in a drying oven (Heraeus W.C., Hanau, Germany) at 180°C. Then, the samples were cooled down at room temperature and vortexed after adding distilled water until a volume of 10 ml was reached. The concentration of the element was determined considering the weight of every single sample and expressed as μg/g wet weight of samples.

For hair cortisol analysis, 100 mg (0.100 ± 0.0005 g) of each hair sample was mixed with 5 ml of methanol (100%) and shaken on a thermomixer (37°C for 24 h). Then, the samples were centrifuged (15 min at 2.500 g), and 2.5 ml of the solution was transferred to another tube and then dried (50–60°C under a stream of air). Afterward, steroids were re-dissolved in 0.5 ml of enzyme immunoassay (EIA) buffer and shaken for 30 min. The solution was transferred to tubes (Bio-Rad Laboratories, Hercules, USA) and stored at −20°C until analysis. In total, 25 μl of the stored sample was analyzed (in duplicate) in a cortisol EIA. This EIA is performed on microtiter plates and utilizes an antibody raised in rabbits against cortisol-3-CMO:BSA and a biotinylated label. Details of the EIA (including cross-reactions of the antibody) can be found in the study by Palme and Möstl (24). The assay was already successfully applied for measuring hair cortisol in ruminants (25, 26).

### Statistical analysis

Data were presented as means and standard errors. Diagnostic graphs and Kolmogorov–Smirnov tests were used to verify assumptions. Ni, Pb, Al, Fe, and cortisol were log10(x+1)-transformed, and the raw data are presented in tables and figures. Changes over time were analyzed using Linear Mixed models. Year (2 levels: 2021 and 2022) and time (3 levels: before grazing, after 1 month, and 2 months of grazing) were included in the models as repeated measures, while animals were included as a random factor. A diagonal structure was chosen as the covariance matrix. Preliminary analysis showed that there were no significant influences of the year or year x time interaction (Supplementary Figure S3). Thus, according to the aim of the study, only the time (i.e., changes during transhumance) was finally included in the models as the main effect. The Sidak method was used for multiple comparisons. Statistical analyses were performed with SPSS 25.0 (SPSS Inc. Chicago, USA) and considered significant at a level of 0.05.

## Results

### Trace elements and heavy metals

**Figure 1:**
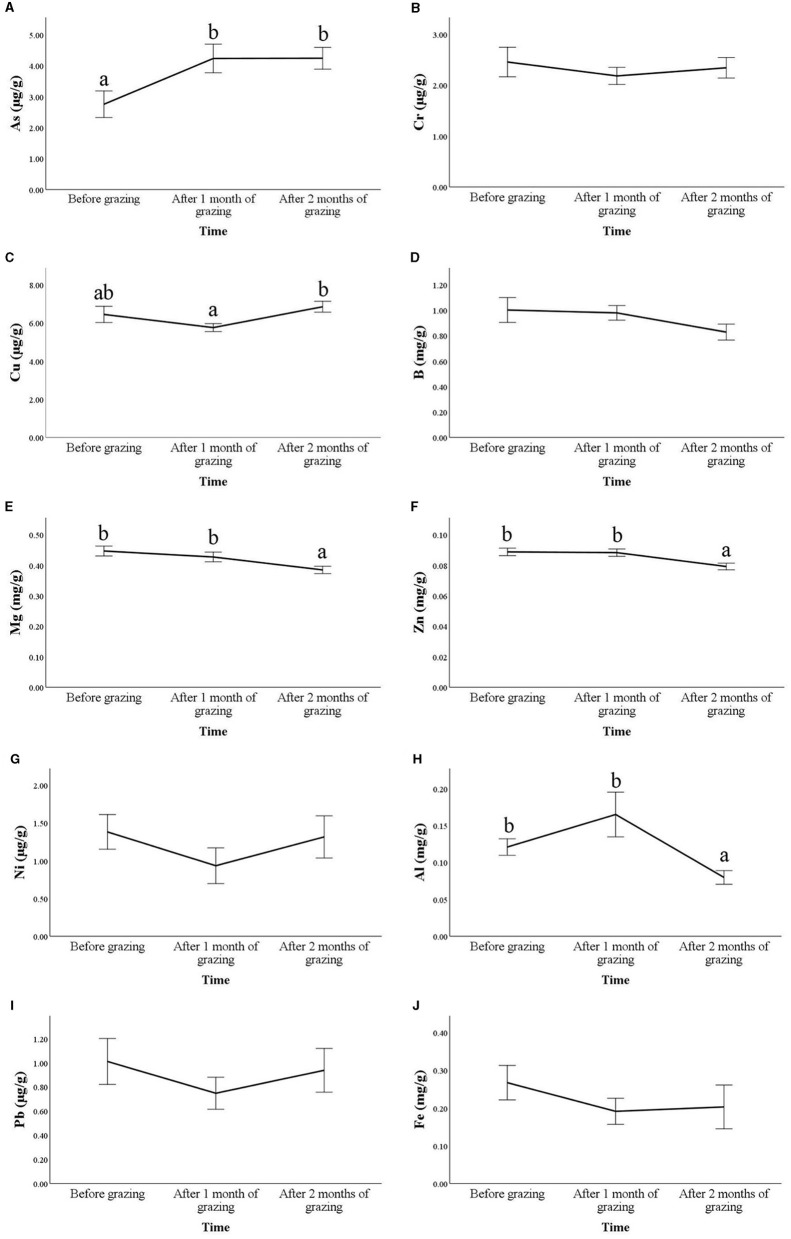
Hair concentrations of trace elements and heavy metals, expressed as μg/g or mg/g of hair, of Frisa goats during vertical transhumance. Hair samples were collected for 2 consecutive years (*n* = 10 in 2021 and *n* = 17 in 2022), 1 day before vertical transhumance (before grazing) and 1 month and 2 months after vertical transhumance. For each molecule, time points that do not share the same letter are different for *p* < 0.05. The time effect was not significant for molecules where no letters are present. The figures show the results for As **(A)**, Cr **(B)**, Cu **(C)**, B **(D)**, Mg **(E)**, Zn **(F)**, Ni **(G)**, Al **(H)**, Pb **(I)**, and Fe **(J)**. Values are means ± standard errors (SE).

Significant changes over time were found for As (*p* = 0.006), Cu (*p* = 0.002), Mg (*p* = 0.002), Zn (*p* = 0.004), and Al (*p* = 0.001; Figure 1).

An increase after 1 month of grazing was found for As, followed by a stabilization in the second month (*p* < 0.05). Cu showed a u-shaped trend where the increase was significant after 2 months of grazing compared with the previous month (*p* = 0.003), but the last time point did not differ from the pre-grazing values (*p* = 0.778).

Conversely, Mg and Zn showed a progressive decrease with significant differences compared with the indoor values only after 2 months of grazing (*p* < 0.05). Al showed highly variable values after 1 month of grazing and a reduction after 2 months compared with pre-grazing (*p* = 0.012).

No significant differences were found among time points for the other heavy metal and trace element concentrations analyzed.

### Cortisol

**Figure 2:**
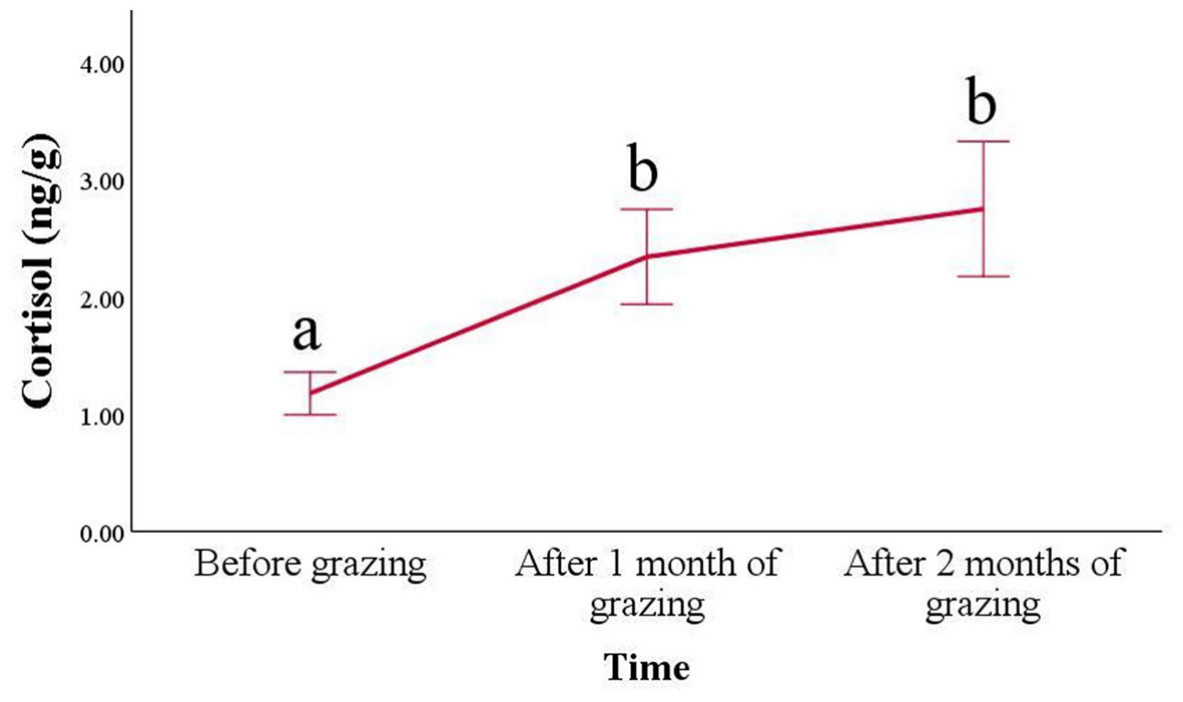
Hair cortisol concentrations (mean ± SE) of Frisa goats during vertical transhumance. Hair samples were collected for 2 consecutive years (*n* = 6 animals in 2021 and *n* = 17 animals in 2022), 1 day before vertical transhumance (before grazing), 1 month, and 2 months after vertical transhumance. Time points that do not share the same letter are different for *p* < 0.05.

Regarding the hair cortisol concentrations (Figure 2), a progressive increase was found in the animals after placing them in the pasture (*p* < 0.001). Its values increased already after 1 month of pasture (2.34 ± 0.41 ng/g) compared with those kept indoors (1.18 ± 0.18 ng/g; *p* < 0.001) and remained higher even after 2 months of grazing (2.75 ± 0.58 ng/g; *p* = 0.004). During grazing, an increase in variability can also be observed.

## Discussion

This study supports the use of the goats' hair as a bioindicator of environmental pollution as its content of some heavy metals and trace elements changed during vertical transhumance in agreement with the change in environment and diet. The findings also showed an increase in hair cortisol concentrations, suggesting activation of the HPA axis linked to the change in the farming system.

As the first aim of our investigation, the results showed an increase in As hair concentration after 1 month of grazing, which was also maintained during the second month. On the other hand, Mg, Zn, and Al concentrations decreased after 2 months of alpine pasture. The appropriateness of using livestock animals to monitor the heavy metal and trace element concentrations in the environment has been demonstrated with different biological matrixes, including blood (27–29), liver and kidney (30), and hair (28, 29, 31). To date, the investigations have covered various animal species. In particular, numerous studies have been conducted on wild animals (32) while, among domesticated animals, most of the studies enrolled cows and sheep (11, 33, 34). Conversely, although the use of goat hair to monitor environmental contamination with heavy metals and trace elements has been validated (26), the literature on this species is scarce. For this reason, there are no reference intervals established for heavy metal and trace element concentrations in goat hair. However, in the present study, the values obtained for Zn, Ni, Pb, and Fe are in line with the available reports (27, 30, 31, 35) while several authors found low er values for As (35–38). It is important to underline that, in any case, the contents of As are much lower than those obtained in experimentally or naturally induced chronic arsenicosis (36–38). The explanation for this result lies in the specific As spatial distribution in Italy. Indeed, the Italian Alps are one of the most As-enriched areas of the peninsula due to their pedogeochemical characteristics. Specifically, Val Bregaglia, which is the breeding area of the goats enrolled in our study, shows As anomalies in agricultural and grazing land soils and in the stream water, which represent the feed sources of the enrolled animals (39). Moreover, considering the high affinity of inorganic As compounds to bind to sulfhydryl groups and the amount of cysteine in the hair structure (40), the high concentration of As in the hair of Frisa goats could be easily explained. The incremental trend in As concentration during alpine pasture is also attributable to the different distribution of As in soil and water in the Lombardy region. In fact, the hay fed to the animals during the period of indoor housing came from Pavia, a province in the Po River plain where the alluvial sediments are also enriched in As, but at a lower level with respect to the Alpine arch soil, as reported by the EuroGeoSurveys GEMAS (Geochemical Mapping of Agricultural and Grazing land Soil) project (39). Regarding Mg, to the best of our knowledge, there are no other studies on goats investigating its concentration in hair. Its values showed a significant decrease after 2 months of alpine pasture. In a study conducted in Switzerland evaluating the spatial distribution of the plant-available forms of Mg in soil, it has been demonstrated that, in general, permanent grasslands had a higher amount of available Mg forms than the alpine pastures. The difference has been ascribed to fertilization with organic manure, which is a common practice in the maintenance of permanent grasslands and enriches the soil with various elements, such as Mg (41). The same difference in land use is present in our study, where the hay fed before vertical transhumance is produced in the permanent grasslands of the Po River valley, while during summer grazing, the only source of feed is the alpine pasture. Regarding Zn, the values obtained in our study were consistent with the scientific literature (27, 35). Its temporal pattern was the same as Mg. In that case, as reported by another investigation on indoor and alpine pasture cow cheese, the variability of Zn content in biological matrixes of animals is subjected to several factors (42), including the soil composition (43), the mineral content of the alpine pasture plants (44), and feeding behavior of the animals (9). For this reason, it is difficult to point out a single explanation for its fluctuations. Finally, Al hair concentration follows the same time trend as Mg and Zn, decreasing after 2 months of alpine pasture. In addition to that case, the major route of entrance in the animal organism is the ingestion of contaminated feed. Different anthropogenic activities are responsible for the Al soil enrichment and consequent increase in the bioavailability of this compound in plants (45). In a study on roe deer hair (32), the animals that fed near urban areas had higher hair concentrations of Al than animals feeding in rural areas (32). In the same way, we could hypothesize that the difference in Al values before and after vertical transhumance could be due to the different origins of the feed. Indeed, the hay administered during indoor housing was from Pavia province, which is greatly urbanized if compared with the alpine pasture area where the goats fed after the transhumance. Interestingly, in the case of Mg, Zn, and Al, it took 2 months to produce any change in the hair of goats, which highlights the necessity of chronic exposure to have detectable changes in the hair matrix. Therefore, for the results obtained from our study, we can confirm that the sampling of hair from autochthonous breeds of livestock could be a valid tool as a bioindicator of environmental contamination by heavy metals and trace elements, with positive implications for the safeguard of humans and environmental health.

According to the One-Welfare approach, however, the wellbeing of humans and the environment cannot be disconnected from that of animals (19, 20). Thus, in addition to the determination of contaminants, the same, non-stressful, hair sampling was exploited to quantify an important physiological indicator of animal welfare, such as cortisol. It also increased the innovativeness of the study because changes in hair cortisol during goat transhumance have not yet been investigated. The results showed a progressive increase in hair cortisol concentration after the vertical transhumance. The values remained high even during the second month of alpine pasture. In goats, the validation of hair cortisol concentrations as an index of long-term activation of the HPA axis was already demonstrated by Endo et al. (22). It must be noted that the activation of the axis is not necessarily linked to negative stimuli and has primarily an adaptive function (described by the classic General Adaptation Syndrome, GAS) (17). Indeed, reproductive and physical activities, positive emotional states, and metabolic processes can induce an increase in the production of cortisol by the adrenal gland (23). Moreover, non-animal factors including environmental and climatic changes as well as the risk of predation, diseases, and social conflicts may result in the activation of the HPA axis as an adaptive response (46). In the present study, several possible biases were eliminated by including only animals of the same sex, age group, physiological status, and coat color. Therefore, mainly the factors linked to transhumance remain as possible explanations for the cortisol changes. To the best of our knowledge, there are no other studies investigating the variations in cortisol concentration in any biological matrixes of goats moving from indoor to outdoor farming systems. However, Comin et al. evaluated hair cortisol concentration variations in cows moving from valley farms to summer pastures (47). They used the same experimental design, and their results are strongly consistent with our findings. Indeed, the hair cortisol concentration increased 1 month after transhumance and remained constant even 2 months after transferring to the cows. The values obtained by these authors are in line with those obtained in the present study, although cows had higher basal hair cortisol concentrations. We hypothesized that the environmental change during transhumance acts *per se* as a stressor [intended as a generic stimulus that requires adjustment or coping strategies (48–50)], determining the activation of the GAS and the HPA axis. The maintenance of high concentrations of cortisol may result from increased physical activity associated with searching for feed sources, particularly on alpine pastures that cover a large area. This enabled the performance of the animal's behavioral repertoire and must therefore be viewed positively in terms of animal welfare. We tend to discard the presence of other specific stressors, such as predators. Indeed, wolf (*Canis lupus italicus*) and bear (both *Ursus arctos marsicanus* and *Ursus arctos arctos*), the only predators in Italy for the adult domestic goat were not present in the considered area (51–53). Parasitosis also seems implausible as the late spring–summer period, during which the experimental trial took place, is characterized by a lowering trend of the parasitic load on the pasture due to the progressive reduction in humidity and increased UV intensity (54). Negative interspecific interactions are also likely to be discarded as a cause of the increased cortisol, as the animals did not mix with other flocks during the alpine grazing period and the space available per head increased considerably as a result of the passage to an extensive system. Our findings also showed a higher variability of hair cortisol concentration during grazing, indicating individual differences in HPA axis reactivity and subjective responses to the change in the farming system.

Finally, it could be mentioned that the effect of year and its interaction with transhumance were not significant for any variable evaluated. This indicates that there have been no relevant changes in heavy metal and trace element concentrations in the areas under study, and the effect of transhumance on the HPA axis has not changed over the years. Several sources of pollution, such as intensive agriculture and industry, can lead to changes in soil contamination, but these are generally evident in medium-to-long-term period (55, 56). Thus, 2 years of monitoring (as carried out in the present study) may be insufficient to highlight the temporal evolution of trace element and heavy metal accumulation. We did not even have a rationale for hypothesizing changes in cortisol concentrations between years. For these reasons, the evaluation of temporal variations was beyond the scope of the present study. However, a longer observation period, or rather, the systematic sampling of animal hair, might be useful for monitoring the time evolution of contaminant distribution and animal welfare.

In conclusion, the close bond between autochthonous livestock breeds, such as goats, and the surrounding environment, where they live, is a factor to be exploited for studies on environmental changes. The hair is a non-invasive matrix that can also provide information on the activation of the HPA axis, the maintenance of homeostasis, and the success of animal adaptation. All these comply with the modern concept of One-Welfare which emphasizes the link between animal welfare, human wellbeing, and the environment. The hair offers practical benefits as well. It is easy to collect and allow repeated measurements over time on the same subject. In addition, this matrix also gives cumulative results, making it possible to eliminate any daily fluctuations, a factor that is particularly important when assessing stress and wellbeing, especially in the case of cortisol. We demonstrated the possible associations between environmental and hair trace element and heavy metal concentrations, specifically regarding the case of As. Moreover, the hair cortisol concentration allowed for the evaluation of the stress response during vertical transhumance, and we showed an increase in the activation of the HPA axis during the first 2 months of the alpine pasture. Future investigation should include a longer period of evaluation for both the hair trace element and heavy metal and hair cortisol concentrations. Moreover, other indicators of stress adaptation and welfare, such as dehydroepiandrosterone, should be evaluated, together with the determination of trace elements and heavy metals in local soil, water, and plants.

## Data availability statement

The original contributions presented in the study are included in the article/Supplementary material, further inquiries can be directed to the corresponding authors.

## Ethics statement

The animal study was approved by the Institutional Animal Care and Use Committee of Università degli Studi di Milano. The study was conducted in accordance with the local legislation and institutional requirements.

## Author contributions

SA: Conceptualization, Data curation, Investigation, Methodology, Visualization, Writing—original draft. AM: Formal analysis, Investigation, Writing—review & editing. OB: Investigation, Methodology, Writing—review & editing. RP: Investigation, Methodology, Writing—review & editing. DT: Investigation, Methodology, Writing—review & editing. BB: Investigation, Methodology, Writing—review & editing. BD: Investigation, Methodology, Writing—review & editing. AE: Investigation, Methodology, Writing—review & editing. MO: Investigation, Methodology, Writing—review & editing. GB: Conceptualization, Funding acquisition, Investigation, Methodology, Project administration, Resources, Supervision, Validation, Writing—review & editing. GC: Formal analysis, Resources, Supervision, Writing—review & editing. SD: Formal analysis, Investigation, Resources, Writing—review & editing. DV: Conceptualization, Funding acquisition, Resources, Supervision, Writing—review & editing. MM: Funding acquisition, Investigation, Writing—review & editing. MG-C: Formal analysis, Investigation, Supervision, Writing—review & editing. LM: Software, Writing—review & editing, Conceptualization, Data curation, Formal analysis.
