# Supplementary material for: Goat hair as a bioindicator of environmental contaminants and adrenal activation during vertical transhumance
Source: Front Vet Sci. 2023 Nov 9;10:1274081. doi: 10.3389/fvets.2023.1274081 (PMC10666633; doi:10.3389/fvets.2023.1274081)
Supplement: Supplementary file 1 [file Data_Sheet_1.pdf]

Supplementary Material

Supplementary Figure 1. Frisa goats grazing on alpine pasture during summer (2021).

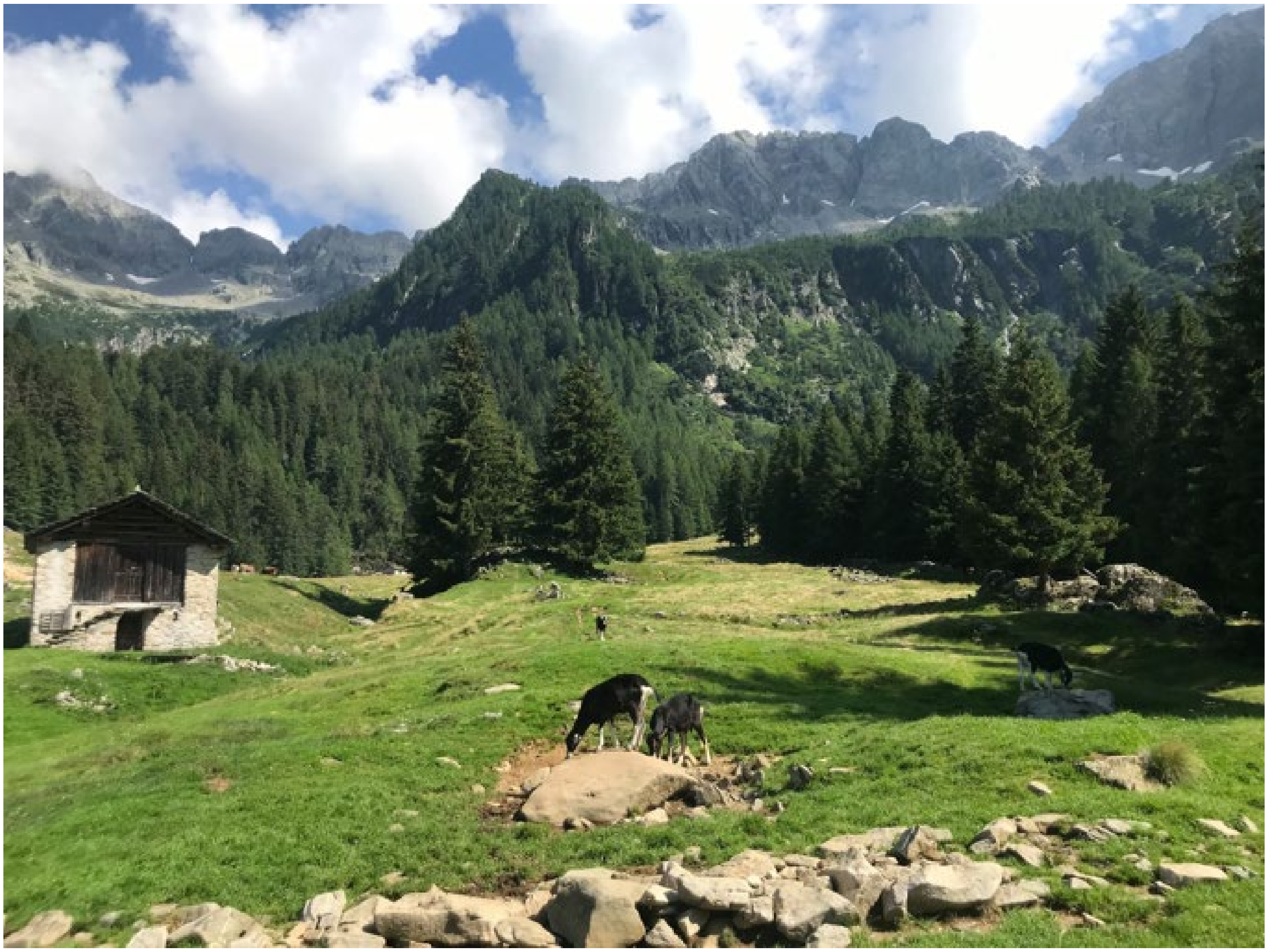

Supplementary Figure 2. Lombardy map showing the area of production of hay fed during winter season (red circle) from Pavia province in the Po River plain, and the area of alpine pasture grazed in summer season (blue circle) in Val Bregaglia (Sondrio province).

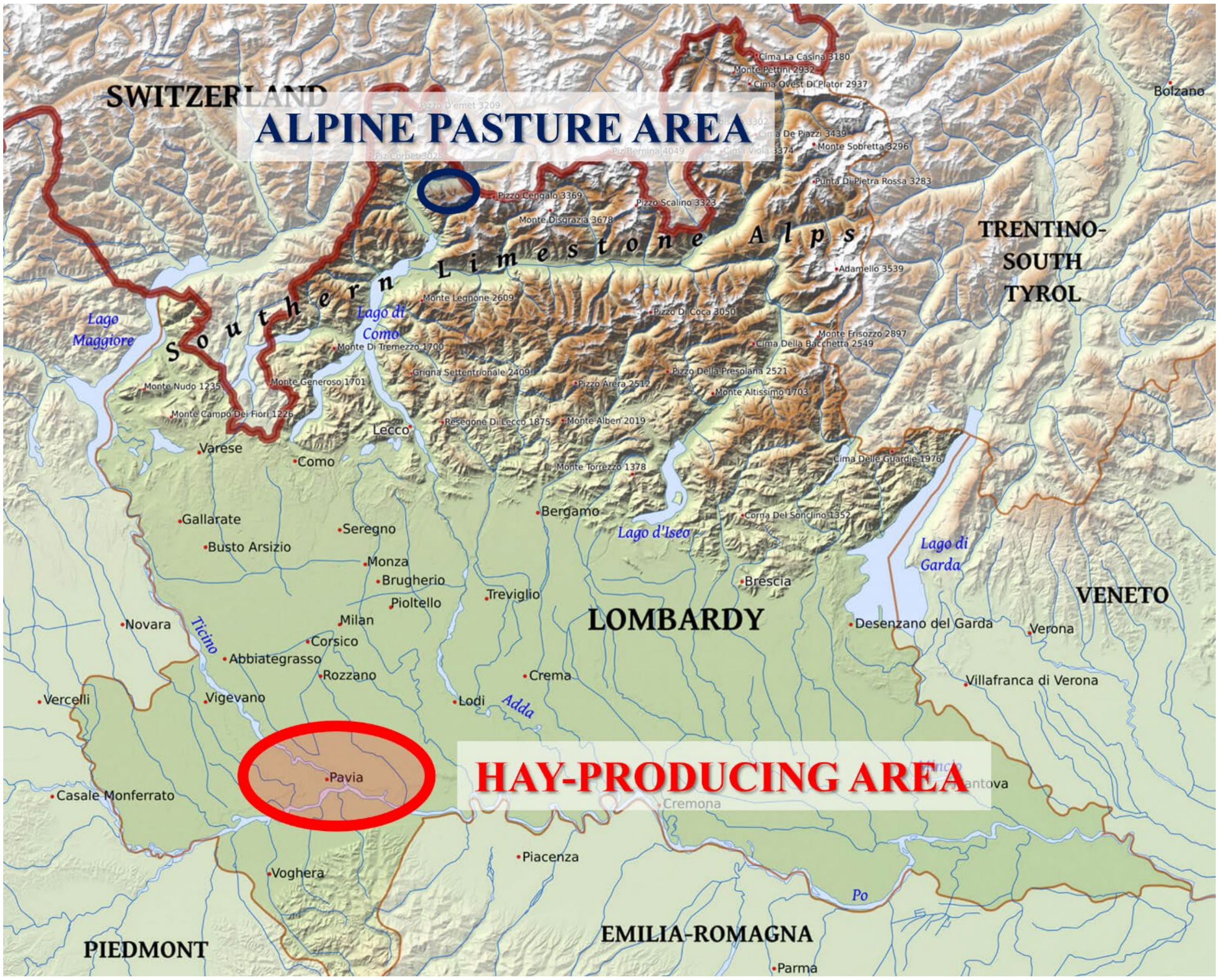

Supplementary Figure 3. Changes in hair concentrations of trace elements and heavy metals, and cortisol during transhumance stratified by year, and results of the factorial models and pairwise comparisons.

**Descriptive statistics and multiple comparisons**	**P value**		
	**Time**	**Year**	**Time x year**
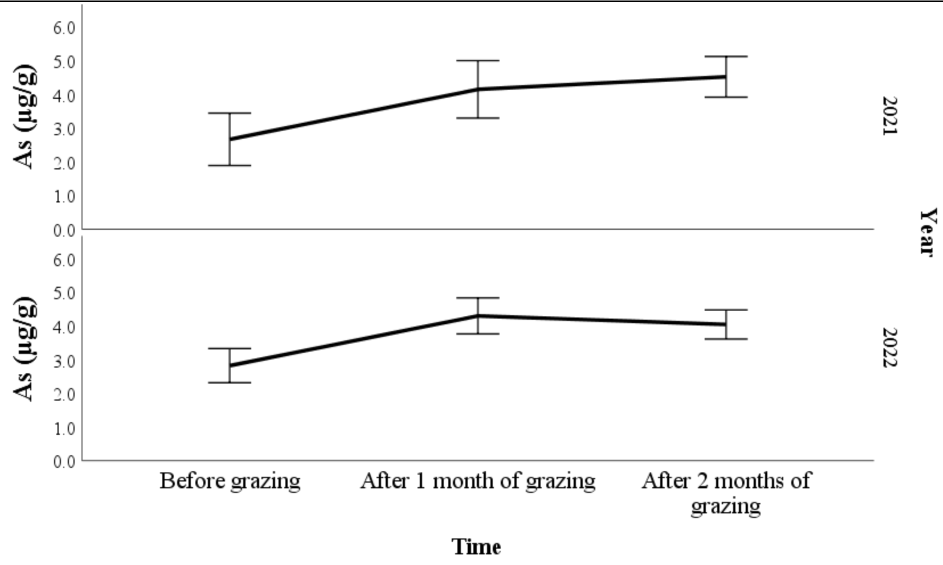	0.014	0.867	0.775
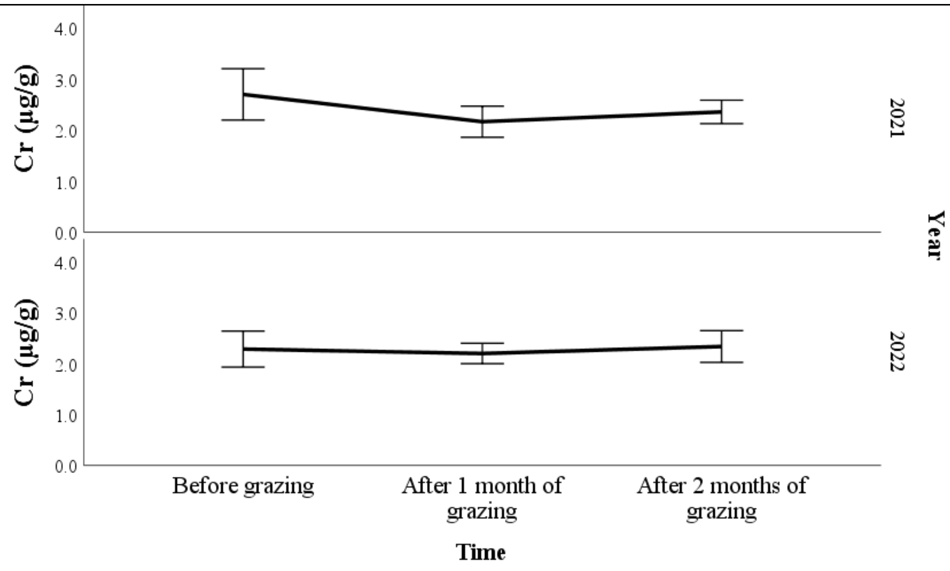	0.602	0.664	0.798

<table>
<tr>
<td>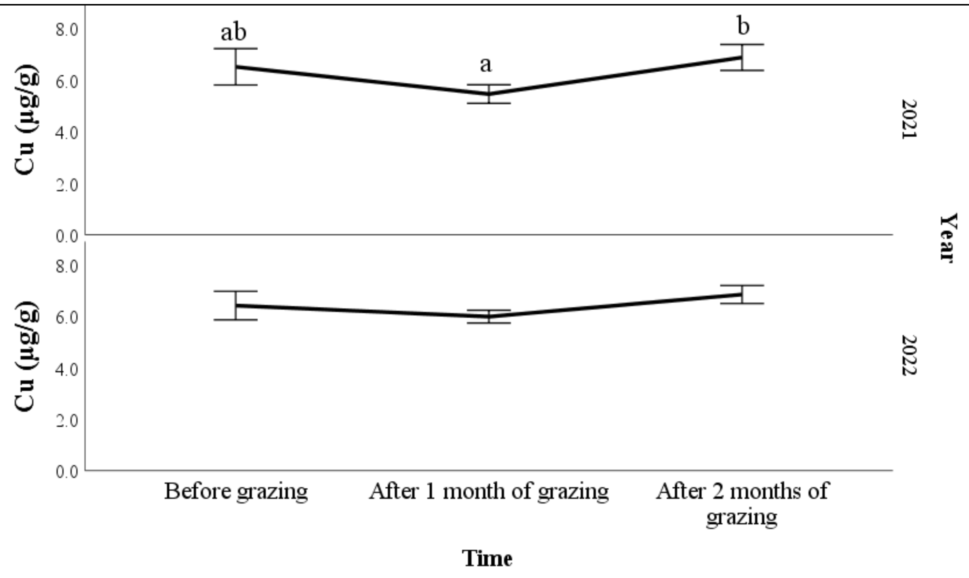
</td>
<td>0.001</td>
<td>0.786</td>
<td>0.539</td>
</tr>
<tr>
<td>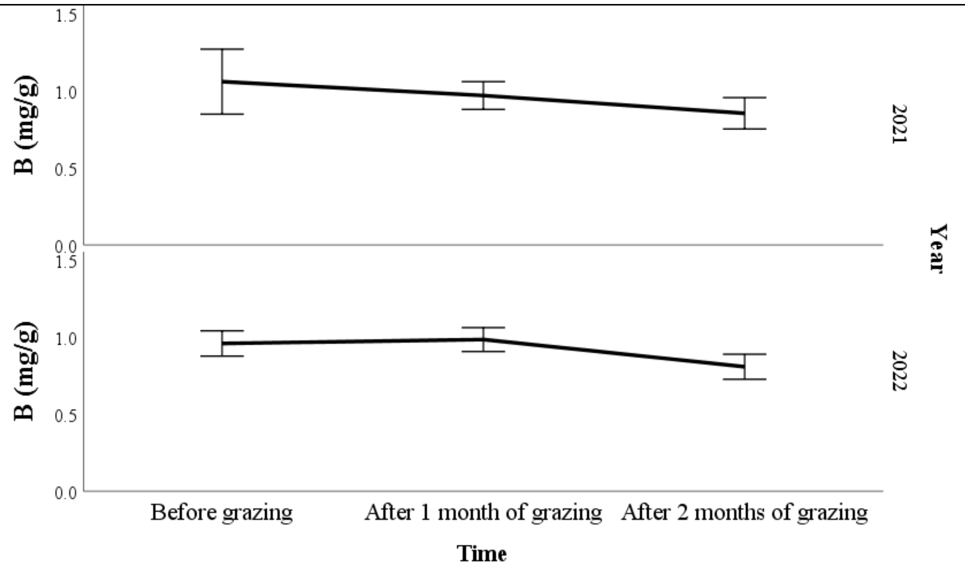
</td>
<td>0.163</td>
<td>0.669</td>
<td>0.865</td>
</tr>
</table>

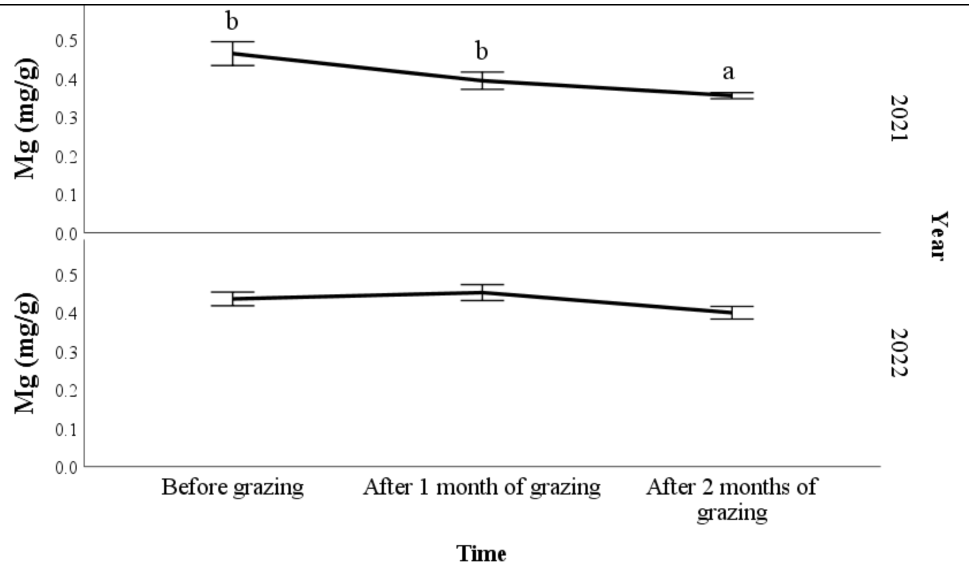	0.001	0.158	0.145
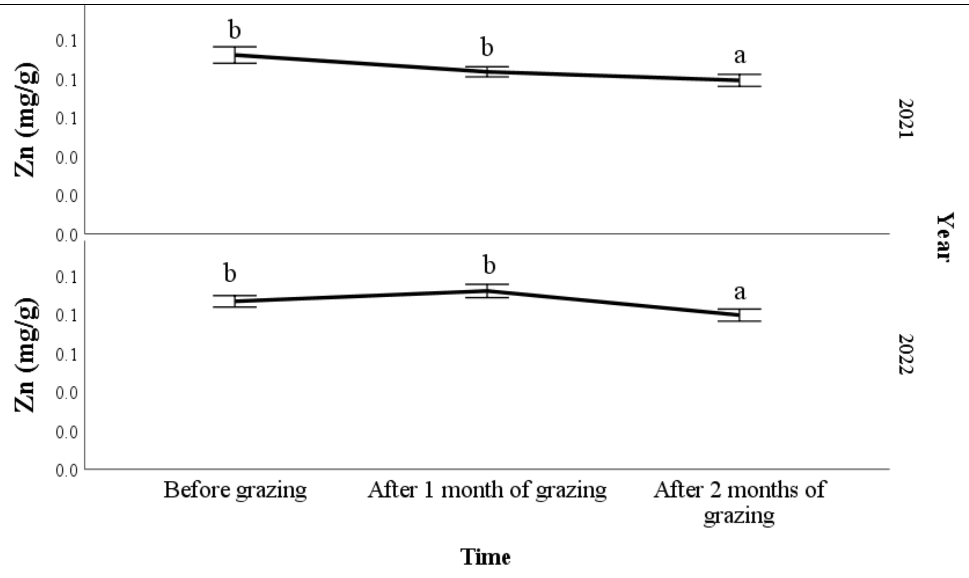	0.002	0.700	0.068

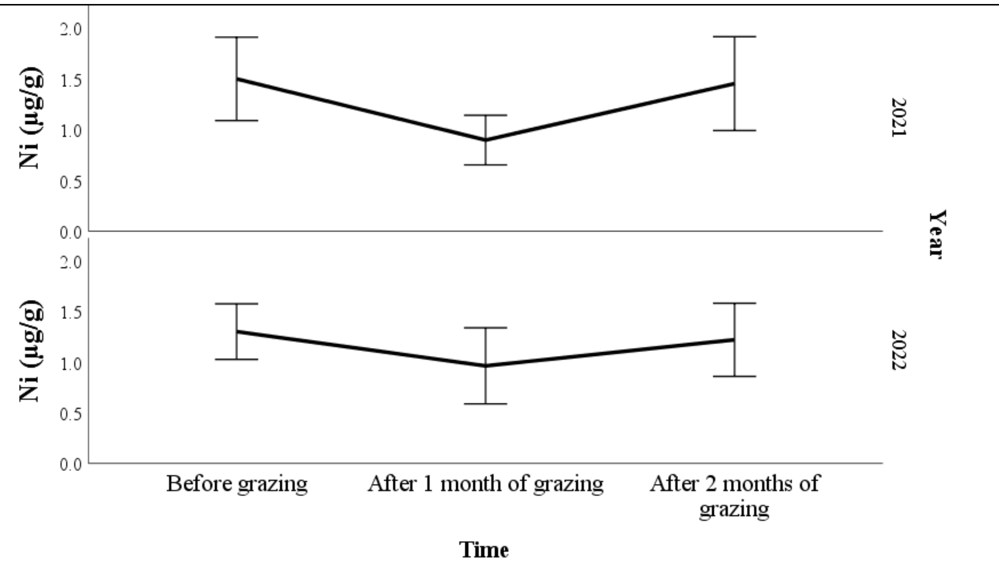	0.161	0.511	0.988
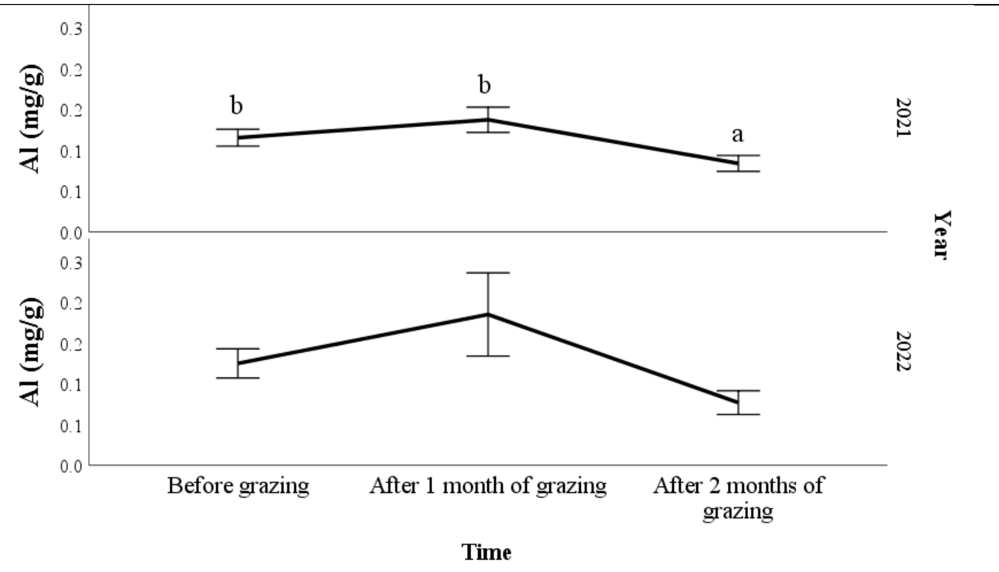	0.001	0.449	0.561

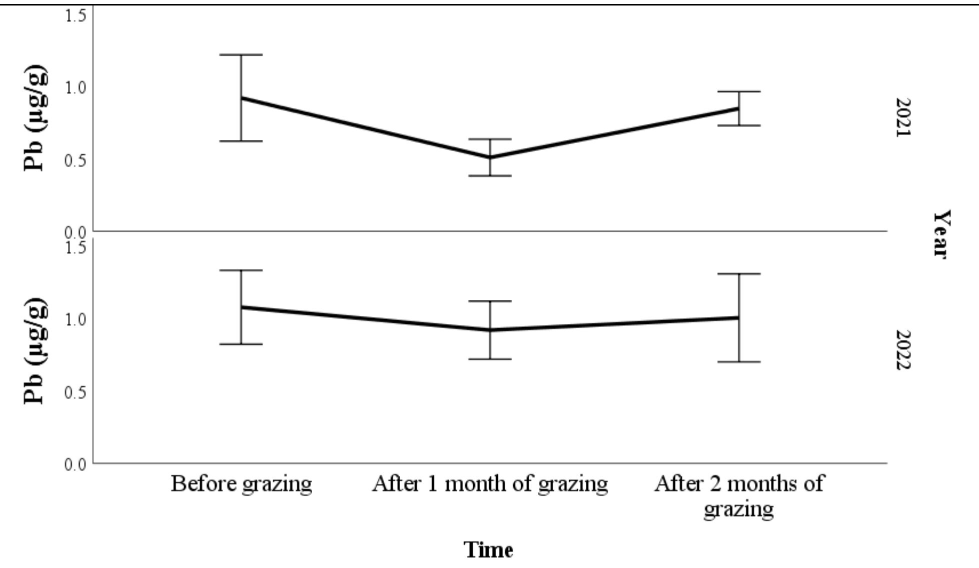	0.412	0.276	0.504
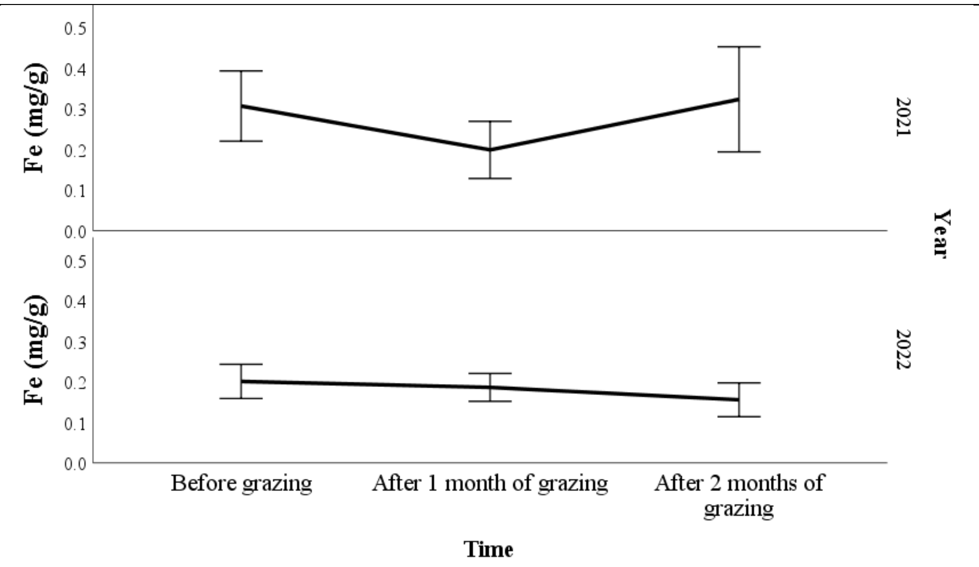	0.524	0.133	0.430

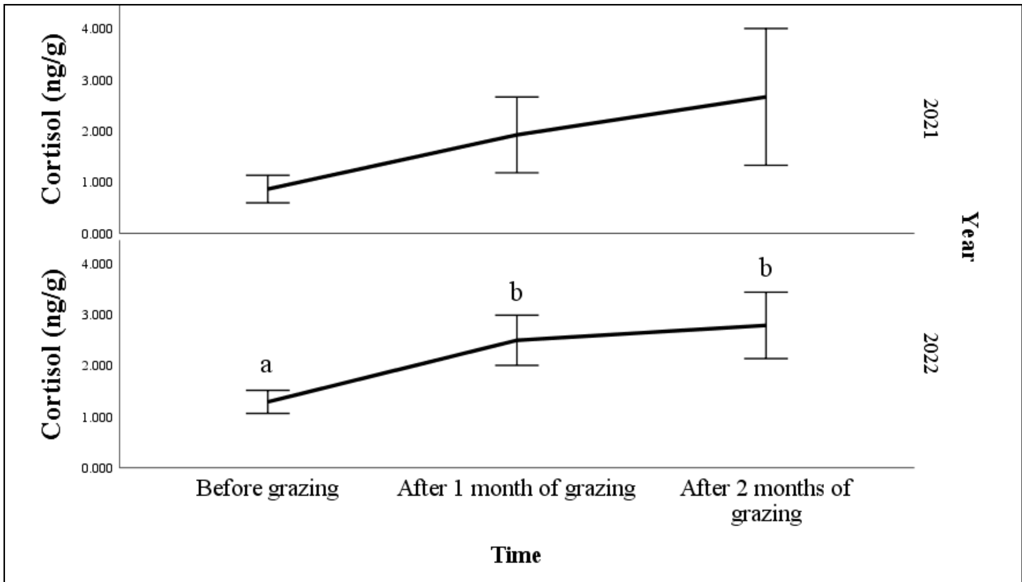	0.010	0.195	0.980

Time points that do not share the same letter are different for p<0.05 (Sidak correction). Pairwise comparisons were not significant for molecules where no letters are present.

Supplementary Table 1. ICP-OES device parameters for determination of trace and toxic elements.

Parameteres	***Assigned value***
Plasma gas flow rate	*15 L/min*
Argon carrier flow rate	*0.5 L/min*
Sample flow rate	*1.51 L/min*
The speed of peristaltic pump	*100 rpm*
RF Power	*1150 W*

Supplementary Table 2. Wave lengths used in the analysis for each element.

Elements	***Wave Length (nm)***
Aluminum (Al)	*167.070*
Arsenic (As)	*189.042*
Boron (B)	*208.959*
Chromium (Cr)	*267.716*
Copper (Cu)	*324.754*
Iron (Fe)	*259.940*
Magnesium (Mg)	*285.213*
Nickel (Ni)	*341.476*
Lead (Pb)	*220.353*
Zinc (Zn)	*206.200*

Supplementary Table 3. Content of the elements and heavy metals assessed in the hair of Frisa goats (n=24) collected one day before vertical transhumance (before grazing), one month, and two months after the vertical transhumance. Values are means and standard errors (SE).

	Time					
	Before grazing		*After 1 month of grazing*		*After 2 months of grazing*	
Parameter	***Mean***	***SE***	***Mean***	***SE***	***Mean***	***SE***
As (µg/g)	*2.75*	*0.43*	*4.23*	*0.46*	*4.24*	*0.35*
Cr (µg/g)	*2.46*	*0.29*	*2.18*	*0.17*	*2.34*	*0.20*
Cu (µg/g)	*6.45*	*0.43*	*5.76*	*0.21*	*6.85*	*0.29*
B (mg/g)	*1.00*	*0.10*	*0.98*	*0.06*	*0.83*	*0.06*
Mg (mg/g)	*0.45*	*0.02*	*0.43*	*0.02*	*0.38*	*0.01*
Zn (mg/g)	*0.09*	*0.00*	*0.09*	*0.00*	*0.08*	*0.00*
Ni (µg/g)	*1.38*	*0.23*	*0.93*	*0.24*	*1.32*	*0.28*
Pb (µg/g)	*1.01*	*0.19*	*0.75*	*0.13*	*0.94*	*0.18*
Al (mg/g)	*0.12*	*0.01*	*0.16*	*0.03*	*0.08*	*0.01*
Fe (mg/g)	*0.27*	*0.05*	*0.19*	*0.03*	*0.20*	*0.06*
